# Caesarean Section Delivery and Risk of Poor Childhood Growth

**DOI:** 10.1155/2020/6432754

**Published:** 2020-04-24

**Authors:** Mahama Saaka, Addae Yaw Hammond

**Affiliations:** University for Development Studies, School of Allied Health Sciences, P.O. Box TL 1883, Tamale, Ghana

## Abstract

**Background:**

Though emerging evidence indicates caesarean section (CS) brings about late initiation of breastfeeding, early cessation of breastfeeding, and a higher risk of developing obesity, little is documented on the association between CS birth and stunted growth. This study assessed caesarean section delivery and the risk of poor postnatal childhood growth.

**Methods:**

A retrospective cohort study design was used to collect the requisite data on a sample of 528 mothers having children between the ages of 6 to 24 months. An interviewer-administered questionnaire was used to collect the data.

**Results:**

After controlling for potential confounding factors, linear growth as measured by height-for-age *Z*-score (HAZ) was significantly higher by 0.121 standard units in children born through normal vaginal delivery, compared to their counterparts born through caesarean section (beta coefficients (*β*) = 0.121, *p*=0.002). The mode of delivery also had a statistically significant impact on infant feeding practices. Whereas 70.4% of babies delivered via vagina initiated breastfeeding within one hour of delivery, only 52.7% of babies born through CS did the same. Vaginally delivered babies were 2.1 times more likely to initiate breastfeeding within one hour of delivery ((Crude odds ratio (COR) = 2.13, *p* < 0.001). Compared to CS babies, vaginally delivered babies were 3.2 times more likely not to have been fed with prelacteal feeds such as water and sugar solutions. Vagina delivered babies were 1.8 times more likely to receive adequate neonatal feeding than their counterparts who were delivered through CS (COR = 1.76, *p*=0.003).

**Conclusions:**

This study has found an association between CS delivery and stunting, an adverse outcome that clinicians and patients should weigh when considering in particular elective CS that seeks to avoid the pain associated with a vaginal birth.

## 1. Background

Stunted child growth or poor linear growth (height for age Z-score 2 standard deviations below the median of the WHO reference population) remains a major public health problem in most places especially in deprived communities [1]. As of 2017, nearly 151 million (22%) children under five years old were stunted [2], and this accounts for 35–45% of worldwide deaths among children less than five years old [1, 3]. By virtue of the adverse consequences of stunted growth, it has been targeted to be reduced globally among children under five by 40% by 2025 [4].

As reported in the 2008 Lancet series on maternal and childhood undernutrition, Ghana was classified among the 36 countries in the world with the highest burden of chronic childhood undernutrition [5]. In Ghana, there has been a slow reduction in stunting rates, where it reduced nationally from 35% in 2003 to 19% in 2014 but the current prevalence of stunting in Northern Region of Ghana which stands at 33% [6] is above internationally acceptable levels.

Global assessment of stunting in low- and middle-income countries revealed that the causes of stunting are multifaceted including poor nutrition, infectious diseases, and household environment [7] and that growth restriction in utero and lack of access to sanitation are the main drivers of stunting [8]. Though a multitude of risk factors has been documented, these vary from place to place and, therefore, context specific. Furthermore, a full understanding of these factors and their relative effect is key to help priority setting in designing policies and interventions to improve childhood growth.

In recent years, another health issue, which experts have expressed worry about, is the rise in caesarean section (CS) childbirths which could have possible negative consequences for maternal and infant nutritional outcomes. CS is an important medical procedure for saving infant and maternal lives in emergency obstetric circumstances and has helped reduce maternal and neonatal morbidity and mortality particularly when medically indicated [9], but it has been on the rise globally. Some health experts have questioned whether CS prevalence beyond recommended levels has any additional benefits to the populace. The most common recommended threshold is the 15% upper limit and 10% lower limit suggested by the World Health Organization (WHO) in 1985.

In Ghana, CS has been on the increase similar to global trends. In 2003, the rate was 3.7%, and as of 2014, it rose to 13%. These levels vary with respect to regions and some recording as high as 23% [6, 10]. A CS rate of 13% is above the lower safe limit of 10% suggested by the WHO and is particularly worrying when juxtaposed with other recommended thresholds such as that of Ye et al. [11], who suggested that the acceptable rate for CS should be 5% to 10% and “caesarean section rates higher than 10% at the population level are not associated with decreases in maternal and neonatal mortality rates.”

When CS is medically indicated, it undeniably saves lives but the high prevalence of low-risk women taking the surgery is unnecessary. It implies that women and neonates are being exposed to avoidable risks as well as increasing the burden of cost on health systems [12].

Though emerging evidence indicates that CS brings about late initiation of breastfeeding, early cessation of breastfeeding, higher risk of developing obesity, among others [13–19], very few studies, if any, have been conducted to assess the association between caesarean section and stunting. In Northern Ghana where stunting rates remain high with the attendant increase in CS deliveries, no study as yet investigated the effects of CS on nutritional outcomes such as growth. It is this knowledge gap that this study sought to explore and fill. To better understand the persisting challenges of reducing stunting, we assessed the prevalence of caesarean section delivery and its relationship with the risk of stunting in childhood. It is hoped that findings from this study will help shed light on and support research-based policy suggestions with a view of addressing problems of the high prevalence of malnutrition amid increasing rates of CS globally.

## 2. Materials and Methods

### 2.1. Study Design, Population, and Sampling

A retrospective cohort study design was used to collect the requisite data. Mothers of children between the ages of 6 to 24 months were selected systematically from six health institutions that provide the Child Welfare Clinic (CWC) services. The attendance registers which served as the sampling frame were used to select two groups of eligible respondents: the nonexposed group comprising women whose index child was delivered without CS and the exposed group comprising women who delivered the index child through CS.

A minimum sample size of 482 (241 per study arm) was needed in order to have an 80% power of detecting a significant difference of 8% in the primary outcome measure between the study groups at 95% confidence interval, and making provision of 10% contingency, the sample size was adjusted to 530. However, 528 mother and child pairs took part in this study which comprised 242 that had caesarean section and 286 mothers having a normal delivery.

### 2.2. Data Collection

A structured pretested questionnaire was used to collect quantitative data. One-to-one interviews were conducted and other vital information was extracted from antenatal records booklet.

### 2.3. Independent and Dependent Variables

The main outcome measure was the low height for age (stunting). The exposure or independent variable was the mode of delivery (that is, caesarean section or normal delivery). A brief description of how some of these variables were measured is as follows.

### 2.4. Dependent Variable: Stunting (Low Height for Age)

Chronic malnutrition (stunting) was measured as low height-for-age Z-scores (HAZ) [20]. The height-for-age Z-score, as defined by the WHO, expresses a child's height in terms of the number of standard deviations above or below the median height of healthy children in the same age group or in a reference group. Stunted children were those having their height-for-age Z-score 2 standard deviations below the median of the WHO reference population.

### 2.5. Potential Confounding Factors

Other potential confounding variables measured in the study were sociodemographic characteristics including the age of child and mother, parity, marital status, religion, educational background of mothers and household wealth index the socioeconomic status of the mother, the nutritional status of the mother measured as body mass index (BMI), the fathers' occupation and occupational status, the mothers' formal educational status, the immunization status of the child, birth weight of the child, the number of antenatal care (ANC) visits before birth, the duration of the pregnancy, APGAR score, the place of delivery, the number of children under five in household and mother's breastfeeding status, duration of breastfeeding, childhood illnesses such as acute respiratory infection during two weeks preceding the study, and household wealth index.

Household wealth index was quantified as a score of household assets such as ownership of means of transport, ownership of durable goods, and household facilities which were weighted using the principal components analysis method [21]. A brief description of the main independent and dependent variables is as follows.

### 2.6. Anthropometric Measurement

Anthropometric measurements of length and weight were obtained following standardized techniques and equipment. The length of the infants (6–23 months) was measured to the nearest 0.1 cm in a recumbent (lying) position using a horizontal wooden length board and movable headpiece (infantometer). The height was measured in older children in standing position. The weight in light clothes was obtained using a digital weighing scale (SECA 890) to the nearest 0.1 kg. Anthropometric indicators of height for age (HAZ), weight for age (WAZ), and weight for height (WHZ) were determined as recommended by the WHO [22].

### 2.7. Data Quality Control Measures

Data collectors and supervisors were trained for two days prior to data collection in line with the objectives. The content of the training included the aim of the study, survey methodology including the selection of eligible participants, data recording, administration of questionnaires, and the art of interviewing and supervision.

The standardization test was carried out during the training to ensure that participants were well equipped with the requisite skills for the anthropometric measurement. The study questionnaire was also pretested and validated in 5% of mothers/caregivers that were not part of the study sample.

### 2.8. Data Management and Analyses

Data cleaning and analysis were carried out using SPSS for Windows 22.0 (SPSS Inc., Chicago).

The Emergency Nutrition Assessment (ENA) for SMART software (2010 version) was used for the anthropometric data analysis and reported using WHO 2006 growth reference values with Standardized Monitoring and Assessment of Relief and Transitions (SMART) cut-offs.

A comparative analysis was carried out between normal vaginal and caesarean delivery on postnatal child growth. We conducted two-step hierarchical multiple regression analyses to determine independent predictors of height for age of children under two years. Multicollinearity was investigated by using the variance inflation factor (VIF). VIF (the reciprocal of the tolerance statistics) of greater than 5 is generally considered evidence of multicollinearity.

Explanatory variables that were significant at bivariate analysis at a *p* value of 0.05 or less were fed into the regression model after confirming the absence of multicollinearity between these independent variables.

### 2.9. Ethics Consideration

The study protocol was approved by the Scientific Review and Ethics Committee of the School of Allied Health Sciences, University for Development Studies, Ghana. The ethics aspects of the study were also reviewed by the same scientific review committee that reviewed the study protocol. Informed written consent was obtained from the literate participants who were also provided copies of the signed forms for their records. In situations, where the participants could not write or read, verbal informed consent was sought after providing the needed information and explanation.

## 3. Results

### 3.1. Sociodemographic Characteristics of Respondents

Five hundred and fifty-eight mother and child pairs took part in this study which comprised 242 (45.8%) that had caesarean section and 286 (54.2%) mothers having a normal vaginal delivery. The mean age of the exposed mothers is 28.65 ± 5.72 while that of the nonexposed group is 26.99 ± 5.89. The mean age (months) of the children for the exposed group is 12.81 ± 4.9 and that of the nonexposed group is 12.00 ± 4.43. More (66.0%) younger (<25 years) respondents had normal delivery while women who had caesarean delivery were more likely to be of the elderly (34 years +). Those with higher education (at least SHS) and high wealth index were more likely to have been exposed to CS, 60.1% and 52.4%, respectively.

At baseline, there were significant differences between the study groups with respect to the age of mother, maternal educational level, the timing of the first ANC visit, and household wealth index (Table 1).

### 3.2. Comparison of Child Growth Indicators according to the Mode of Delivery

This study aimed to determine whether caesarean delivery is a risk factor for stunting in children aged 6–24 months. The child's growth rate (g/month) was calculated based on the following formula: growth rate = current weight (g)–birthweight (g)/age (months). The mean child's growth rate was 455.1 ± 158.2 (g/month) among children aged 6–24 months.

The results show that caesarean delivery was associated negatively with the height-for-age *Z*-score of the child but not with the mean birth weight and weight-for-height *Z*-score. Children who were born through caesarean section (CS) had lower mean HAZ, compared to their counterparts who were born normally through the vagina (Table 2). There was no significant association between caesarean delivery and the other growth indicators, i.e., birth weight (g), weight for height, weight for age, and growth rate.

When the height-for-age Z-scores were treated as a categorical variable, the prevalence of stunted growth was still significantly higher among children born through caesarean delivery (49.2 versus 31.1%) (Chi-squared = 17.9, *p* < 0.001).

### 3.3. Predictors of Height-for-Age Z-Score (HAZ): Multivariable Regression Analysis

After controlling for potential confounding factors, children who were born through normal vaginal delivery had 0.121 standard units higher HAZ than their counterparts born through caesarean section (*p* < 0.0001).  Table 3 shows the “coefficients” table with the predictors which were statistically significant.

Considering the beta coefficients (*β*), children of at least third birth order had mean HAZ which was 0.491 standard units significantly lower than their counterparts who were first born (beta = [−0.491 (95% CI: −1.11 to −0.44)). A unit increase in the age of child led to reduced HAZ of 0.206 standard units. The strongest predictor of mean HAZ was parity of the mother (>2) with a standardized beta (*β*) weight of 0.391, *p* < 0.001. The second highest contributor was the birth weight of the child with beta (*β*) weight = 0.356, *p* < 0.001. A unit increase in maternal height led to increased HAZ of 0.245 standard units (beta (*β*) = 0.245 (95% CI: 0.04, 0.07)).

Female children had a mean HAZ that was significantly higher by 0.133 standard units (beta *β* = 0.245, *p*=0.001) and children born to women aged at least 35 years had a higher mean HAZ of 0.129 standard units, compared to children born to women under 25 years (beta *β* = 0.129, *p*=0.004). The set of variables accounted for 26.1% of the variance in mean HAZ (adjusted *R*-squared = 0.261).

Using the hierarchical multiple regression approach, the covariate predictor variables (main effects) were entered in the first step. In the second step, the main explanatory variable of interest (that is, the mode of delivery) was added.

The percentage of variability in the dependent variable that can be accounted for by all the predictors together is measured by R-squared. The change in *R*^2^ is a way to evaluate how much predictive power was added to the model by the addition of another variable. In step 2 when the main explanatory variable was added to the model, the percentage of variability accounted for went up from 25.8% to 27.2% (*R*^2^ Change = 0.014, *p* < 0.001).

### 3.4. Relationship between Mode of Delivery and Neonatal Feeding Practices

To characterize infant feeding practices, we computed a composite infant feeding index (IFI) comprising current breastfeeding status of the child, timely initiation of breastfeeding within one hour, bottle feeding in the past 24 hours, prelacteal feeding, the introduction of complementary foods at 6 months, and feeding colostrum to the child.

Each positive practice/health behaviour was assigned a score of 1; otherwise, a score of zero was assigned. For example, a child receiving colostrum was a positive practice and so a score of 1 was given. Children who were bottle-fed or received prelacteal feed was score zero. A child meeting 5–6 of these practices was described as having appropriate infant feeding, and scores less than 5 criteria were classified as having inappropriate infant feeding.

The mean infant feeding index (IFI) for infants born through normal vaginal delivery was significantly higher than that for infants born through CS (5.0 versus 4.7) (*F* (1,527) = 10.55, *p* = 0.001).

From Table 4, the results showed that the mode of delivery was associated with the overall infant feeding index, timely initiation of breastfeeding within one hour, and prelacteal feeding. Infants delivered by caesarean section were less likely of being fed appropriately.

Whereas 70.4% of babies delivered via vagina initiated breastfeeding within one hour of delivery, only 52.7% of babies born through CS did the same. Vagina delivered infants were 2.1 times more likely to practice this behaviour. Compared to CS babies, vaginally delivered babies were 3.2 times more likely not to have been fed with prelacteal feeds such as water and sugar solutions. Vagina delivered babies were 1.8 times more likely to receive adequate neonatal feeding than their counterparts who were delivered through CS (COR = 1.76, *p*=0.003).

### 3.5. Factors Associated with Mode of Delivery

As shown in Table 5, older women of at least 25 years were more likely to have caesarean delivery (CS). Women of the high educational level of at least Senior High School (SHS) were more likely to have caesarean delivery. Women who initiated ANC early within the first trimester of pregnancy were more likely to have caesarean delivery. CS operation was more common among women whose baby's birth weight was less than 2.5 kg, compared to babies of weight at least 2.5 kg (63.2% versus 42.4%, *p* < 0.001). Caesarean delivery was also common in households of high wealth index and women who had previous CS were more likely to have a delivery through CS. Overweight and obese women were prone to CS delivery.

Multivariable logistic regression analyses showed that systolic BP at 36 weeks of gestation, birth order, educational level of the mother, maternal height, mother's age, and having obstetric abnormalities during pregnancy were factors contributing significantly to caesarean section delivery (Table 6). Compared to mothers who initiated antenatal care beyond the first trimester, a greater proportion of women who initiated ANC in the first trimester had caesarean delivery (OR = 1.98, CI = 1.10–3. 55).

Compared to third-order babies, first- and second-order babies were 2.3 and 2.4 times more likely of being delivered through caesarean section (AOR = 2.29 and 2.39, respectively).

The data showed that a unit increase in maternal height led to 9% protection against CS delivery (AOR 0.91; 95% CI (0.87–0.95)). Women who had obstetric abnormalities during pregnancy were 2.7 times more likely to have CS (AOR 2.68; 95% CI (1.39–5.16)), compared to their counterparts who had no such problems.

Caesarean section rates tend to be higher among more educated women (AOR 2.94; 95% CI (1.49–5.84)).

The set of predictors accounted for 29.5% of the variation in the mode of delivery (Nagelkerke *R*-squared = 0.295).

## 4. Discussion of Results

We investigated the association between caesarean section (CS) birth and the risk of undernutrition among children aged 6–24 months. The main finding was that after controlling for potential confounding factors, children who were born through normal vaginal delivery had 0.121 higher HAZ than their counterparts born through caesarean section (*p* < 0.0001). However, results show no significant association between caesarean delivery and the other growth indicators such as birth weight, weight for height, weight for age, and growth rate.

### 4.1. The Association between CS and Child Growth Indicators

Of all the nutritional status indicators assessed (i.e. height for age, weight for height, weight for age, and growth rate), it was only low height for age (stunting) that was significantly associated negatively with CS. Since the set of variables accounted for only 26.1% of the variance in mean HAZ, this suggests that other confounders not known or not accounted for could influence child growth. This together with the cross-sectional nature of the study means that only associations, and not causation, are indicated.

Stunting remains a major public health concern in many countries including Ghana as it has negative consequences including increasing the risk of illness, irreversible body damage, and mortality in children. Public health planners will have to consider effective interventions to reduce stunting by having full knowledge of its determinants. It is against this backdrop that our finding that CS deliveries associate with stunting is relevant to the programme planning and collaboration among all practitioners (that is, clinicians, policymakers, public health experts, etc.)

### 4.2. Potential Mechanisms Linking Mode of Delivery and Stunting

The study findings suggest that there is an association between caesarean delivery (CD) and stunting in children though underlying biological mechanisms still remain unclear. However, the mode of delivery may adversely affect child growth through its influence on feeding practices. In the present study, we found that CD was associated negatively with infant feeding practices including timely initiation of breastfeeding and prelacteal feeding. For example, vagina-delivered infants were 2.1 times more likely to initiate breastfeeding within one hour of delivery.

Breastfeeding infants within one hour of birth ensures that the infant receives the colostrum, or “first milk,” which is rich in protective factors. This is the rationale behind the WHO recommending that all mothers should be supported to initiate breastfeeding as soon as possible after birth, within the first hour after delivery [23]. If the child's immune system is compromised, then the child becomes vulnerable to infections that may affect growth and development. Many other studies have reported that CS birth negatively affects timely initiation of breastfeeding [13–18, 24, 25] and that late initiation of breastfeeding after one hour after birth is significantly associated with stunting [26].

There is also a growing body of evidence that demonstrates the importance of early establishment and diversity of gut microbiota for the prevention of risks in the offspring. It has been reported that babies delivered by CS are generally not exposed to their mother's vaginal and faecal microbiota and that the gut microbiome of such babies has low diversity and richness [27, 28]. This microbiota helps to shape the initial composition of an infant's microbiota including that of the gut [27]. Findings from some other studies suggest that infants born by CS might have a gut microbiota that has a tendency to harvest more dietary nutrients, thereby predisposing them to increased risk of being overweight or obese [29, 30], and that persists through early adult life [31]. Malamitsi-Puchner at al. [32] reported that only vaginal delivery is associated with the production of cytokines implicated in neonatal immunity. This reduced immunity with children born through CS could account for the observed association with stunting.

During normal delivery, the contact with mothers' vagina serves as an important flora for the infant's gastrointestinal tract (GIT) colonization. But during CS, this direct contact is absent; hence, nonmaternally derived bacteria play a vital role in the intestinal colonization [33, 34]. Studies have suggested that the composition of the very first flora in an infant's GIT could have long-term consequences on infants, especially in breastfed infants.

Furthermore, there is epidemiologic evidence of an association between CS birth and increased body mass index (BMI) [35–37]. It must, however, be noted that the evidence for a causal relationship between CS birth and the subsequent risk for obesity and other health risks is not conclusive because there is still no compelling evidence to support a causal link between elective CS and childhood obesity [38].

It appears that both the nonphysiological start of GIT colonization by flora and absence of early dietary support caused by delayed initiation of lactation could account for the long-term effects on the nutrition of children including stunting. However, long-term prospective studies need to be carried out to establish the clinical relevance and impact of this association.

In this present study, maternal short stature, obesity, and low birth order were key determinants of CS delivery and these together can greatly influence stunting either prenatally or postnatally. For example, compared to third-order babies, first- and second-order babies were 2.3 and 2.4 times more likely of being delivered through caesarean section (AOR = 2.29 and 2.39, respectively). Compared to women aged under 25 years, women who were at least 35 years had 4.871 odds of delivery through CS. It is, therefore, possible that some of these factors could jointly be influencing stunting indirectly through CS. From our results, some factors associated with CS were also independent predictors of low HAZ. For example, we found that the risk of C-section was elevated in short women compared to women of normal stature and shorter maternal height was also a risk factor for low HAZ.

### 4.3. Predictors of Height-for-Age Z-Score (HAZ)

After controlling for potential confounding factors, children who were born through normal vaginal delivery had 0.121 higher HAZ standard units than their counterparts born through caesarean section (*p* < 0.0001). The most consistent factors associated with low height-for-age Z-score (HAZ) were the mode of delivery (caesarean section), low birth weight, increasing child's age, sex of child (male), parity of the mother (<2), mother's age (<25 years), low mother's height, and increasing birth order.

A search on the literature shows that very few studies have investigated the association between CS and child growth and at least two studies have collaborated the present finding that there is a significant association between stunting and mode of delivery [39, 40].

As it has been reported in previous studies from different countries, the order of birth was one of the significant predictors of the child being stunted in this study population [41–44]. Children of at least third-order birth were more likely to be stunted than children of the first-order birth. A possible explanation for this association could be that food and other essential resources decrease with an increasing number of births in the household, thereby making it impossible to meet essential basic needs including adequate feeding and health care. Consistent with other studies, utilization of antenatal and postnatal care services is also reported to decrease with the higher-order birth [45–47], implying preventive health services are unlikely to be available for such children. The finding in this study and many others, therefore, suggests reducing the number of births and for that matter birth order may reduce child stunting.

Female children had a mean HAZ that was significantly higher than their male counterparts. This means that boys were more likely than girls to have low height for age (stunting), which is consistent with earlier studies reporting similar trends from sub-Saharan Africa [42, 48, 49]. The vulnerability of boys to stunted growth might be explained by the fact that boys are expected to grow at a slightly more rapid rate compared to girls and their growth is perhaps more easily affected by nutritional deficiencies or other diseases or exposures [50].

A unit increase in the age of child led to reduced HAZ of 0.206 standard units and was consistent with similar studies where increasing the age of the child was associated positively with stunting [39, 51–53].

One other key determinant of stunting was low birth weight as the results showed that children born with a low weight (<2.5 kg) were more likely to be stunted, in comparison to children with weight ≥2.5 kg at birth. This finding is consistent with reports from other countries including Ghana, Pakistan, Mexico, and Nepal [39, 54–56] and justifies the need to support interventions that seek to prevent intrauterine growth retardation, premature delivery for the prevention of stunting.

We also found that low maternal height below 145 cm (that is, short stature) increased the risk of low HAZ (stunting). This confirms earlier research findings that high maternal height [52, 57, 58] is positively associated with improved child nutrition.

Children born to women aged at least 35 years had a higher mean HAZ compared to children born to women under 25 years. This finding is consistent with studies previously conducted in many countries including Nepal Pakistan and Mexico [39, 55, 59–61].

## 5. Conclusions and Recommendations

This study has found an association between CS delivery and stunting (low height-for-age z-scores), an adverse outcome that clinicians and patients should weigh when considering caesarean birth that is not medically indicated to help improve mother and child postnatal health outcomes.

Since timely initiation of breastfeeding rate was significantly higher among women who had vaginal birth compared with those who had CS, it is highly recommended that postcaesarean practices such as rooming-in and skin-to-skin contact should be promoted in all health delivery facilities.

### 5.1. Limitations of the Study

This study was retrospective in nature and so responses provided depended on the mother's recall memory which can introduce measurement bias. The cross-sectional nature of the study also makes it difficult to demonstrate cause-and-effect relationships. Notwithstanding this, we have provided important insights into the relationship between caesarean birth and stunting.

## Figures and Tables

**Table 1:** Comparison of sociodemographic characteristics by study groups (*N* = 528).

Variable		*N*	Study groups		Test statistic
			Caesarean delivery	Normal delivery	
			*n* (%)	*n* (%)	
Age of mother (years)					
Under 25		162	55 (34.0)	107 (66.0)	*χ* ^*2*^ = 13. 3, *p*=0.001
25–34		306	157 (51.3)	149 (48.7)	
35+		60	30 (50.0)	30 (50.0)	
Maternal education					
None		184	66 (35.9)	118 (64.1)	*χ* ^*2*^ = 27.2, *p* < 0.001
Low (primary & JHS)		141	54 (38.3)	87 (61.7)	
High (at least SHS)		203	122 (60.1)	81 (39.9)	
Marital status					
Married		513	237 (46.2)	276 (53.8)	*χ* ^*2*^ = 0. 9, *p*=0.3
Single		15	5 (33.3)	10 (66.7)	
Religion				
Islam		494	224 (45.3)	270 (54.7)	*χ* ^*2*^ = 0. 7, *p*=0.4
Christianity		34	18 (52.9)	16 (47.1)	
Trimester of first ANC visit				
1–3 months		367	180 (49.0)	187 (51.0)	*χ* ^*2*^ = 5. 7, *p*=0.02
>3 months		159	60 (37.7)	99 (62.3)	
Frequency of ANC visits				
Less than 4		8	5 (62.5)	3 (37.5)	*χ* ^*2*^ = 0. 9, *p*=0.3
At least 4		512	233 (45.5)	279 (54.5)	
Classification of parity				
Primiparous		160	72 (45)	88 (55.0)	*χ* ^*2*^ = 2. 0, *p*=0.4
Secundiparous		161	81 (50.3)	80 (49.70)	
Multiparous		207	89 (43.0)	118 (57.0)	
Classification of wealth index				
Low		280	112 (40.0)	168 (60.0)	*χ* ^*2*^ = 8. 2, *p*=0.004
High		248	130 (52.4)	118 (47.6)	
Employment status				
Unemployed		112	45 (40.2)	67 (59.8)	*χ* ^*2*^ *=* 1.8, *p*=0.2
Employed		416	197 (47.4)	219 (52.6)	

**Table 2:** Comparison of child growth indicators according to mode of delivery.

Categories of maternal height (cm)	Child growth indicator	*N*	Mean	Std. Deviation	95% confidence interval for mean		Test statistic
					Lower bound	Upper bound	
Vaginal delivery	Birth weight of child (g)	286	2967.34	502.03	2908.91	3025.77	*F* (1, 524) = 1.628, *p*=0.20
Caesarean delivery		242	2906.78	588.78	2832.22	2981.33	
Vaginal delivery	Height for age of child	283	−1.47	1.26	−1.62	−1.32	*F* (1, 524) = 10.569, *p*=0.001
Caesarean delivery		242	−1.84	1.36	−2.01	−1.67	
Vaginal delivery	Weight for height of child	282	−0.59	1.12	−0.72	−0.46	*F* (1, 524) = 2.047, *p*=0.15
Caesarean delivery		241	−0.45	1.15	−0.59	−0.30	
Vaginal delivery	Weight for age of child	286	−1.45	1.13	−1.58	−1.32	*F* (1, 524) = 0.988, *p*=0.32
Caesarean delivery		242	−1.55	1.21	−1.71	−1.40	
Vaginal delivery	Growth rate of child in grams/month	286	459.23	148.26	441.97	476.48	*F* (1, 524) = 0.416, *p*=0.52
Caesarean delivery		242	450.31	169.46	428.85	471.76	

**Table 3:** Predictors of height-for-age Z-score (HAZ).

Model	Standardized coefficients			95.0% confidence interval for *β*		Collinearity statistics	
	Beta	T	Sig.	Lower bound	Upper bound	Tolerance	VIF
(Constant)		−9.326	<0.001	−16.42	−10.71		
Gender of child	0.134	3.446	0.001	0.152	0.56	0.959	1.042
Height of mother	0.256	6.680	<0.001	0.042	0.08	0.985	1.015
Birth weight	0.359	9.098	<0.001	0.680	1.06	0.931	1.075
Age of child	−0.215	−5.567	<0.001	−0.08	−0.04	0.972	1.029
Mothers' age	0.106	2.441	0.015	0.04	0.41	0.763	1.310
Parity	0.371	3.419	0.001	0.25	0.93	0.123	8.104
Birth order	−0.457	−4.229	<0.001	−1.057	−0.39	0.124	8.055
(Constant)		−9.254	<0.001	−16.20	−10.525		
Gender of child	0.133	3.440	0.001	0.15	0.55	0.959	1.042
Height of mother	0.245	6.401	<0.001	0.04	0.07	0.976	1.025
Birth weight	0.356	9.097	<0.001	0.68	1.05	0.930	1.075
Age of child	−0.206	−5.365	<0.001	−0.08	−0.04	0.966	1.035
Mothers' age	0.129	2.932	0.004	0.09	0.46	0.743	1.346
Parity	0.391	3.626	<0.001	0.29	0.96	0.123	8.133
Birth order	−0.491	−4.555	<0.001	−1.11	−0.44	0.123	8.137
Mode of delivery (vaginal)	0.121	3.108	0.002	0.12	0.52	0.949	1.054

**Table 4:** Relationship between the mode of delivery and infant feeding practices.

Infant feeding practice	Mode of delivery		Crude odds ratio COR (CI)
	Caesarean	Vaginal	
	*n* (%)	*n* (%)	
Timely initiation of breast feeding within one hour	116 (52.7)	183 (70.4)	2.13 (1.46–3.10), *p* < 0.001
No bottle feeding in the past 24 hours	201 (83.1)	237 (82.9)	0.9 (0.63–1.56), *p*=0.9
No prelacteal feeding	207 (87.7)	272 (95.8)	3.2 (1.58–6.37), *p*=0.001
Introduction of complementary foods at 6 months	150 (62.0)	188 (65.7)	1.18 (0.08–1.03), *p*=0.06
Child received colostrum	239 (98.8)	274 (95.8)	0.29 (1.62–7.90), *p*=0.002
Adequate neonatal feeding	150 (62.0)	212 (74.1)	1.76 (1.21–2.55), *p*=0.003

**Table 5:** Factors associated with mode of delivery (*N* = 528).

Factor	*N*	Mode of delivery		
		Caesarean delivery *n* (%)	Vaginal delivery *n* (%)	
Age of mother (years)				
Under 25	162	55 (34.0)	107 (66.0)	Chi-squared (χ^2^) = 13. 3, *p*=0.001
25–34	306	157 (51.3)	149 (48.7)	
35^+^	60	30 (50.0)	30 (50.0)	
Maternal education				
None	184	66 (35.9)	118 (64.1)	*χ* ^2^ = 27. 2, *p* < 0.001
Low (primary & JHS)	141	54 (38.3)	87 (61.7)	
High (at least SHS)	203	122 (60.1)	81 (39.9)	
Classification of household wealth index				
Low (<median score)	280	112 (40.0)	168 (60.0)	*χ* ^2^ = 8.2, *p*=0.004
High (at least median score)	248	130 (52.4)	118 (47.6)	
Had obstetric abnormalities during pregnancy				
No	400	165 (41.3)	235 (58.8)	*χ* ^2^ = 13. 9, *p* < 0.001
Yes	128	77 (60.2)	51 (39.8)	
Adequacy of ANC visits				
No	157	61 (38.9)	96 (61.1)	*χ* ^2^ = 4. 3, *p*=0.04
Yes	363	177 (48.8)	186 (51.2)	
Timing of first ANC visit				
1–3 months	367	180 (49.0)	187 (51.0)	*χ* ^2^ = 5. 7, *p*=0.02
>3 months	159	60 (37.7)	99 (62.3)	
Birth weight				
≥2.5 kg	441	187 (42.4)	254 (57.6)	*χ* ^2^ = 12. 7, *p* < 0.001
<2.5 kg	87	55 (63.2)	32 (36.8)	
Previous caesarean section				
No	456	170 (37.3)	286 (62.7)	*χ* ^2^ = 98. 5, *p* < 0.001
Yes	72	72 (100.0)	0 (0.0)	
Maternal BMI				
Underweight	22	7 (31.8)	15 (68.2)	*χ* ^2^ = 15. 2, *p*=0.002
Normal	285	114 (40.0)	171 (60.0)	
Overweight	160	85 (53.1)	75 (46.9)	
Obese	58	36 (62.1)	22 (37.9)	

**Table 6:** Predictors of caesarean section delivery.

	Wald	Sig.	Exp(B)	95% C.I.for EXP (*β*)	
				Lower	Upper
Systolic BP at 36 weeks	7.523	0.006	1.02	1.01	1.04
Maternal height	15.278	<0.001	0.91	0.87	0.95
Birth order (reference: >3)	7.322	0.026			
First order	5.082	0.024	2.29	1.11	4.71
Second order	6.168	0.013	2.40	1.20	4.79
Timing of first ANC visit in first trimester	5.162	0.023	1.98	1.10	3.55
Educational level of mother (reference: none)	10.754	0.005			
Low (primary and JHS)	0.591	0.442	1.33	0.65	2.71
High (at least SHS)	9.558	0.002	2.94	1.49	5.84
Had obstetric abnormalities during pregnancy	8.637	0.003	2.68	1.39	5.16
Mother's age (reference: under 25 years)	11.706	0.003			
25–34 years	8.800	0.003	2.594	1.382	4.870
At least 35 years	8.780	0.003	4.871	1.709	13.880
Constant	6.472	0.011	14034.008		

## Data Availability

The quantitative data presented in the form of SPSS used to support the findings of this study are included within the supplementary information files.

## Abbreviations

ANC: Antenatal care
AOR: Adjusted odds ratio
BMI: Body mass index
CI: Confidence interval
COR: Crude odds ratio
CS: Caesarean section
CWC: Child Welfare Clinic
ENA: The Emergency Nutrition Assessment
GIT: Gastrointestinal tract
HAZ: Height-for-age Z-score
IFI: Infant feeding index
SMART: Standardized Monitoring and Assessment of Relief and Transitions
WAZ: Weight-for-age Z-score
WHZ: Weight-for-height Z-score
WHO: World Health Organization.

## Supplementary Materials

Supplementary Materials The supplementary materials submitted along with the manuscript were actually the data set supporting the findings.
